# Exploring the Psychological and Physiological Effects of Operating a Telenoid: The Preliminary Assessment of a Minimal Humanoid Robot for Mediated Communication

**DOI:** 10.3390/s24237541

**Published:** 2024-11-26

**Authors:** Aya Nakae, Hani M. Bu-Omer, Wei-Chuan Chang, Chie Kishimoto, Hidenobu Sumioka

**Affiliations:** 1 Presence Media Research Group, Hiroshi Ishiguro Laboratories, Deep Interaction Laboratory Group, Advanced Telecommunications Research Institute International (ATR), 2-2-2 Hikaridai, Seika-Cho, Soraku-Gun, Kyoto 619-0288, Japan; hbuomer@atr.jp (H.M.B.-O.); chou@atr.jp (W.-C.C.); kishimotochie@atr.jp (C.K.); sumioka@atr.jp (H.S.); 2 Global Center for Medical Engineering and Informatics, Graduate School of Medicine, Osaka University, 2-2 Yamadaoka, Suita City 565-0871, Japan; 3 Laboratory of Science & Innovation for Pain, Graduate School of Frontier Biosciences, Osaka University, 2-2 Yamadaoka, Suita City 565-0871, Japan

**Keywords:** avatar, Telenoid, stress, conversation, new acquaintance, cortisol, SRS-18, d-ROMs

## Abstract

Background: As the Internet of Things (IoT) expands, it enables new forms of communication, including interactions mediated by teleoperated robots like avatars. While extensive research exists on the effects of these devices on communication partners, there is limited research on the impact on the operators themselves. This study aimed to objectively assess the psychological and physiological effects of operating a teleoperated robot, specifically Telenoid, on its human operator. Methods: Twelve healthy participants (2 women and 10 men, aged 18–23 years) were recruited from Osaka University. Participants engaged in two communication sessions with a first-time partner: face-to-face and Telenoid-mediated. Telenoid is a minimalist humanoid robot teleoperated by a participant. Blood samples were collected before and after each session to measure hormonal and oxidative markers, including cortisol, diacron reactive oxygen metabolites (d-ROMs), and the biological antioxidat activity of plasma (BAP). Psychological stress was assessed using validated questionnaires (POMS-2, HADS, and SRS-18). Results: A trend of a decrease in cortisol levels was observed during Telenoid-mediated communication, whereas face-to-face interactions showed no significant changes. Oxidative stress, measured by d-ROMs, significantly increased after face-to-face interactions but not in Telenoid-mediated sessions. Significant correlations were found between oxytocin and d-ROMs and psychological stress scores, particularly in terms of helplessness and total stress measures. However, no significant changes were observed in other biomarkers or between the two conditions for most psychological measures. Conclusions: These findings suggest that cortisol and d-ROMs may serve as objective biomarkers for assessing psychophysiological stress during robot-mediated communication. Telenoid’s minimalist design may help reduce social pressures and mitigate stress compared to face-to-face interactions. Further research with larger, more diverse samples and longitudinal designs is needed to validate these findings and explore the broader impacts of teleoperated robots.

## 1. Introduction

Recent advancements in humanoid robots have opened up new opportunities for enhancing human communication through mediated interactions. These robots are being used increasingly frequently in various fields, such as education, healthcare, and customer service, where they act as intermediaries between humans. However, most research in this field focuses on how robots affect the people with whom they interact, rather than on how operating the robots affects the operators themselves. Operating a teleoperated robot involves unique cognitive and physiological demands, which may have implications for both immediate and longer-term operator health. Understanding these effects is critical as teleoperated systems become more integrated into professional and personal contexts [1,2,3].

The rapid expansion of the Internet of Things (IoT) has fundamentally transformed communication styles, with avatars and digital devices becoming integral to daily interactions. This shift is especially evident in the younger generation, who frequently engage in avatar-based communication within virtual worlds, such as online gaming and social media [4]. Mediated communication, such as video conferencing and avatar-based platforms, introduces stressors distinct from face-to-face communication. These include an increased cognitive load from managing interfaces, the unpredictability of partner responses, and the absence of direct social presence. For instance, recent studies have highlighted the phenomenon of ‘Zoom fatigue’, where prolonged engagement in virtual communication leads to physical and psychological exhaustion [5]. Similarly, text-based communication has been reported to induce higher stress levels compared to face-to-face interactions [6]. Despite the growing prevalence of such communication methods, limited research has explored how these interactions impact the operators’ physiological and psychological well-being [7].

In this study, we aim to bridge this gap by exploring the effects of operating a tele-operated robot, specifically Telenoid, on the operator’s psychological and physiological states. Unlike many telecommunication devices, Telenoid’s minimalist design intentionally removes human-like characteristics, such as overt facial expressions, to reduce biases and focus on core interactions. This design makes Telenoid an ideal subject for isolating the effects of robot operation on operators’ physiological and psychological states. By focusing solely on the operator’s interaction with their conversation partner, we can isolate the physiological and psychological responses triggered by the task of operating the robot itself. While cognitive load is an important dimension within understanding operator experiences, this study prioritized stress-related measures, such as hormonal and oxidative markers, to explore the interplay between psychological and physiological responses during robot-mediated communication. Specifically, we investigate changes in hormonal levels and oxidative stress markers, such as diacron-reactive oxygen metabolites (d-ROMs) and biological antioxidant potential (BAP), which have been identified as sensitive indicators of stress and antioxidant responses [8,9]. Hormonal markers like cortisol are well-established indicators of stress, particularly in response to novel or demanding tasks [10,11]. Similarly, oxidative stress markers such as d-ROMs and BAP are becoming increasingly recognized in healthcare research for their ability to reflect short-term physiological changes associated with stress and emotional regulation [12,13]. These markers provide valuable insights into acute stress responses, complementing traditional psychological evaluations to create a comprehensive understanding of the operator’s health and psychological state.

The integration of psychological assessments with physiological measures further strengthens the current study’s methodology. By including validated psychological instruments, such as the Profile of Mood States 2nd Edition (POMS-2) and the Stress Response Scale (SRS-18), we provide a comprehensive view of how the operation of a teleoperated robot influences both the mind and the body. POMS-2 is widely used for assessing transient mood states and has been validated for use in both clinical and research settings, making it an appropriate tool for evaluating mood changes in this study [14,15]. The SRS-18, developed in Japan, is specifically designed to measure stress responses over short periods and has demonstrated good reliability and validity [13,14]. To complement the use of these instruments, this study also considers how novelty may influence psychological and physiological responses, particularly during a person’s initial exposure to teleoperated systems.

By focusing on operators’ physiological and psychological responses during robot-mediated communication, this study adds to the growing body of human–robot interaction research. It highlights the importance of operator-centered evaluations in the design of robots that minimize cognitive and emotional demands. As teleoperated systems increasingly permeate both professional and personal domains, understanding their effects on operators is essential for improving user experience and well-being. These findings can inform future robot design and operation protocols, potentially leading to the development of interventions to mitigate stress and enhance performance during robot-mediated interactions.

## 2. Methods

### 2.1. Study Design

This study was conducted in accordance with the principles of the Declaration of Helsinki and was approved by the Ethics Review Committee of Osaka University Graduate School of Frontier Biosciences (Application Number: FBS2022-1, Approval date: 8 June 2022). To ensure data privacy, all personal data collected during the study were anonymized and securely stored on institutional servers, accessible only to authorized researchers directly involved in the study. Consent forms and other physical records were kept in a locked cabinet. Data will be retained for up to five years, after which electronic records will be securely erased, and physical records will be shredded in compliance with Osaka University’s institutional data protection policies.

The study employed a within-subject design to compare the physiological and psychological effects of two communication modes: face-to-face interactions and communication mediated through a teleoperated robot (Telenoid). Telenoid, a 50 cm long and 3.5 kg portable teleoperated minimalist humanoid robot, was used in this study. Telenoid’s design follows a minimalist anthropomorphic strategy to eliminate the influence of perceived personality traits (e.g., gender, age, appearance) during telecommunication [4]. The head and lip movements of Telenoid were controlled by the operator’s voice using a speech-driven motion system [16]. Telenoid’s facial expression remained unchanged, but its arms moved slightly to mimic involuntary breathing movements. Its tactile quality, made of soft vinyl chloride, was designed to resemble human skin.

Participants completed two communication sessions, one face-to-face with an assistant (Facing session) and one mediated through operating Telenoid (Telenoid session). The order of the sessions was counterbalanced across participants to avoid order effects. The assistants were unfamiliar to the participants, of the opposite sex, and not always the same across sessions to ensure variability in interaction partners. Psychological questionnaires (POMS-2, HADS, SRS-18) were administered both before and after each session to evaluate pre- to post-session changes.

A total of 36 interpersonal questions, drawn from Aron et al.’s interpersonal closeness protocol [17], were used to standardize the content and emotional depth of the conversations. These open-ended questions encouraged participants to discuss personal and varied topics, ensuring consistent interaction across conditions. Participants answered the questions, while the assistants served solely as conversational partners. Each session lasted approximately two hours, with scheduled breaks to minimize fatigue and maintain concentration.

Before and after each conversation session, blood samples were collected to assess changes in physiological and biological markers, including cortisol, d-ROMs, and BAP levels. These data were complemented by psychological measures obtained through validated questionnaires to provide comprehensive insights into changes in both their physiological states and psychological well-being throughout the experiment.

**Figure 1:**
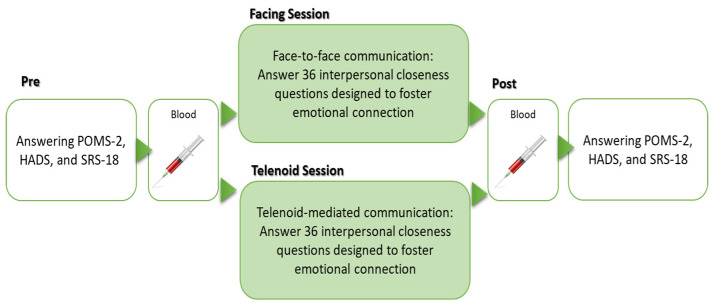
Study design.

**Figure 2:**
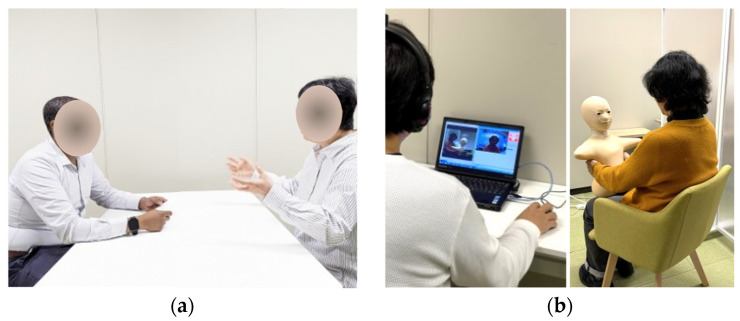
Depiction of experimental setup in the two sessions: (**a**) Facing session and (**b**) Telenoid session.

This design allowed us to evaluate psychological and physiological changes under two distinct communication conditions while controlling for individual variability through a within-subject approach. Figure 1 illustrates the study procedure and Figure 2 presents the experimental setup for both conditions.

### 2.2. Participants

Twelve healthy participants (two females and ten males, aged 18–23 years, M = 21.5) were recruited voluntarily via advertisements on Osaka University’s intranet and through word of mouth. All participants were in good physical and mental health and were able to read and write Japanese without difficulty. Participants were provided with a detailed briefing about the study objectives, procedures, and potential risks. They gave written informed consent before participation and were explicitly informed of their right to withdraw from the study at any time without penalty. No adverse events or harm to participants were reported during the study.

To ensure unbiased results, the sessions’ orders were counterbalanced: five participants began with the Facing session, and seven started with the Telenoid session. Data from all twelve participants were included in the statistical analysis.

### 2.3. Hormonal and Oxidative Stress Marker Measurements

Serum levels of cortisol, dehydroepiandrosterone-sulfate (DHEA-S), and growth hormone (GH) were determined using enzyme immunoassays (EIAs) (cortisol: Detect X Cortisol Enzyme Immunoassay Kit, Arbor Assays, Ann Arbor, MI, USA; DHEA-S: DHEA-S ELISA RUO, DRG International, Inc., Springfield, NJ, USA; GH: Quantikine ELISA Human Growth Hormone Immunoassay, R&D Systems, Inc., Minneapolis, MN, USA). The limits of detection were 45.5 pg/mL (0.00455 μg/dL) for cortisol, 0.044 μg/mL for DHEA-S, and 2.10 pg/mL for GH, with intra- and inter-assay coefficients of variation below 10% for all assays.

Plasma levels of oxytocin (OT) were measured using a competitive immunoassay (Oxytocin ELISA kit, Enzo Life Sciences Inc., Farmingdale, NY, USA) with a detection limit of 5.11 pg/mL and intra- and inter-assay coefficients of variation below 17%.

Serum levels of the oxidative stress markers (d-ROMs) and antioxidant marker (BAP) were measured using d-ROMs and BAP test kits (Wismerll, Tokyo, Japan) and the REDOXLIBRA measuring device. The detection limits for d-ROMs were 11 U. CARR (1 U CARR = 0.08 mg H_2_O_2_/dL), while for BAP it was 150 μmol/L, with intra- and inter-assay variations below 3% for d-ROMs and 4% for BAP.

### 2.4. Mood and Mental Status Assessment Questionnaires

#### 2.4.1. Profile of Mood States (POMS-2)

POMS-2 was used to assess mood states, including anger–hostility (AH), confusion–bewilderment (CB), depression–dejection (DD), fatigue–inertia (FI), tension–anxiety (TA), and vigor–activity (VA) [14]. Total mood disturbance (TMD) was calculated by summing negative mood states and subtracting the positive mood state (vigor). Friendliness (F) was considered a separate factor. The Japanese version of POMS-2, used in this study, has shown sound reliability (Cronbach’s α = 0.84–0.95) and validity [15]. Permission to use the instrument was obtained by purchasing it from the marketer.

#### 2.4.2. Hospital Anxiety and Depression Scale (HADS)

HADS was used to assess participants’ anxiety and depression levels. HADS comprises two subscales: the HADS for anxiety (HADS-A) and the HADS for depression (HADS-D). Both HADS-A and HADS-D consist of seven items and each item is rated on a 4-point Likert scale, with possible total scores ranging from 0 to 21. Anxiety/depression was defined as a HADS-A/HADS-D score ≥8. Anxiety and depression severity were categorized as intact (0–7), mild (8–10), moderate (11–14), or severe (15–21) based on total scores [18,19].

#### 2.4.3. Stress Response Scale (SRS-18)

The SRS-18, a validated Japanese scale, measures the psychological stress encountered over a short period and comprises 18 items related to daily changes in feelings about the events that a typical person experiences [20,21]. It has three subscales, each with six items: depression/anxiety (D/A), hostility/anger (H/A), and helplessness. The items are rated on a 4-point Likert scale (0 to 3), with higher total scores indicating greater stress levels. The internal consistency of the scale is adequate (Cronbach’s α = 0.82–0.88). The minimum and maximum total scores are 0 and 54, respectively, with subscale scores ranging from 0 to 18. Permission to use the scale was obtained by purchasing it from the marketer.

### 2.5. Statistical Analysis

Statistical analyses were conducted using JMP V.17.0 software. Paired *t*-tests were used to compare pre- and post-session changes in cortisol, d-ROMs, BAP, and psychological measures (POMS-2, HADS, SRS-18) within each condition (Facing vs. Telenoid). Given the number of comparisons, a False Discovery Rate (FDR) correction [22] was applied to control for Type I errors while maintaining an appropriate balance between sensitivity and specificity.

FDR correction was chosen over Bonferroni correction due to its less conservative nature, making it more suitable for this exploratory study. The corrected *p*-values were calculated, and the significance level was set at *p* < 0.05 after FDR adjustment. Additionally, *p*-values between 0.05 and 0.1 were considered to indicate a trend or tendency toward significance, which may warrant further investigation in future studies with larger sample sizes. All *p*-values reported in the results are FDR-corrected unless otherwise specified.

Spearman’s rank correlation coefficient (Spearman *ρ*) was used to evaluate the relationships between changes in psychological and physiological markers across conditions. The results are reported in the figures as means ± SEM for each variable.

## 3. Results

### 3.1. Measurements of Hormonal and Oxidation/Antioxidation Markers 

#### 3.1.1. Cortisol Levels

**Figure 3:**
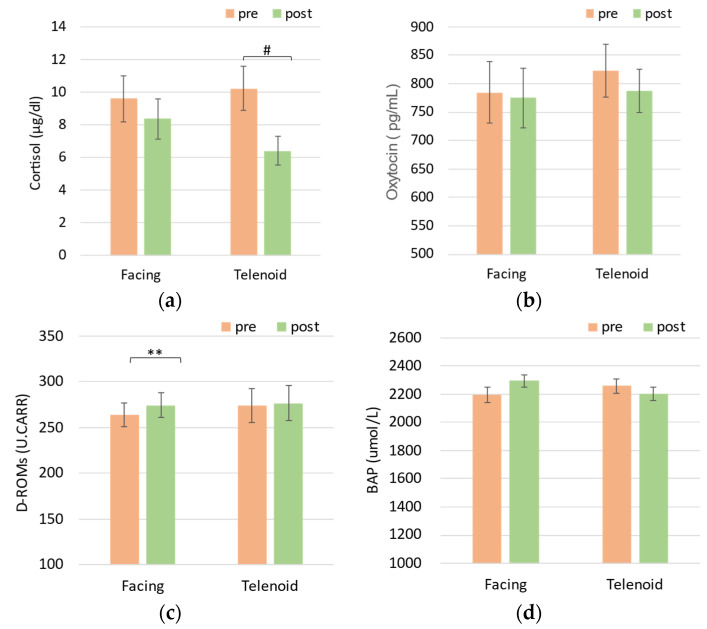
Changes in serum hormones and markers of oxidation/antioxidation levels; serum levels of (**a**) cortisol, (**b**) oxytocin, (**c**) D-ROMs, and (**d**) BAP, before (pre) and after (post) conversation in the Facing and Telenoid sessions. Significant differences indicated as # *p* < 0.1 or ** *p* < 0.01.

Figure 3a illustrates the changes in serum cortisol levels between the direct (Facing) and Telenoid-mediated (Telenoid) communication conditions. Cortisol levels did not significantly change from pre- to post-session in the Facing condition, *t*(11) = 1.11, *p* = 0.402. In the Telenoid condition, cortisol levels showed a trend toward reduction, *t*(11) = 2.57, *p* = 0.067, suggesting a potential decrease post-session. The difference between the two conditions was not statistically significant: *t*(11) = 1.60, *p* = 0.226.

#### 3.1.2. Oxytocin Levels

Figure 3b shows the changes in oxytocin levels across both the Facing and Telenoid conditions. No significant changes were observed in oxytocin levels in the Facing condition, *t*(11) = 0.48, *p* = 0.6945, or the Telenoid condition, *t*(11) = 2.03, *p* = 0.1337. Additionally, the difference between the two conditions was not significant: *t*(11) = 1.11, *p* = 0.3963.

#### 3.1.3. d-ROMs Levels

Figure 3c shows the changes in d-ROMs levels across the two conditions. A significant increase in d-ROMs levels was observed from pre- to post-session in the Facing condition, *t*(11) = −4.97, *p* = 0.008, while no significant change was detected in the Telenoid condition, *t*(11) = −0.92, *p* = 0.452. The difference between the two conditions did not reach significance: *t*(11) = 2.07, *p* = 0.162.

#### 3.1.4. BAP and Other Hormones

Figure 3d presents the changes in BAP levels across the two conditions. No significant changes were found in BAP levels in either the Facing condition, *t*(11) = −1.47, *p* = 0.275, or the Telenoid condition, *t*(11) = 1.45, *p* = 0.275. The difference between the conditions was also not significant: *t*(11) = 1.91, *p* = 0.178. The changes in the growth hormone and DHEA-S responses were also not significant in either session.

### 3.2. Psychological Questionnaire Results

#### 3.2.1. POMS-2 Results

**Figure 4:**
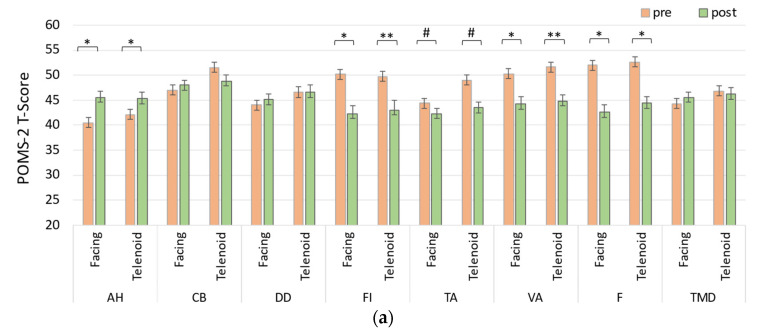

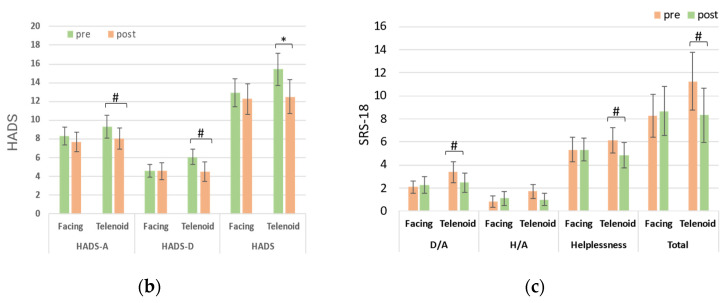
Changes in the scores of each questionnaire before (pre) and after conversation (post) in the Facing and Telenoid sessions: (**a**) POMS-2, (**b**) HADS, and (**c**) SRS-18. Significant differences are indicated as # *p* < 0.1, * *p* < 0.05, or ** *p* < 0.01.

Figure 4a illustrates the changes in POMS-2 subscale scores across both the Facing and Telenoid conditions. In the Facing condition, anger–hostility (AH) showed a significant increase, *t*(11) = −3.36, *p* = 0.0231. Conversely, significant decreases were observed in the fatigue–inertia (FI) subscale, *t*(11) = 3.97, *p* = 0.0132, the friendliness (F) subscale, *t*(11) = 4.33, *p* = 0.0108, and the vigor–activity (VA) subscale, *t*(11) = 3.73, *p* = 0.0151. Additionally, the tension–anxiety (TA) subscale showed a trend toward a decrease, *t*(11) = 2.36, *p* = 0.0762.

In the Telenoid condition, AH also showed a significant increase, *t*(11) = −4.03, *p* = 0.0132. Significant decreases were noted in the FI subscale, *t*(11) = 4.69, *p* = 0.0079, the F subscale, *t*(11) = 3.75, *p* = 0.0151, and the VA subscale, *t*(11) = 5.27, *p* = 0.0076. A trend toward a decrease was observed for TA, *t*(11) = 2.86, *p* = 0.0510.

When comparing the two conditions, no significant differences were found between the two conditions (Facing vs. Telenoid) for any of the POMS-2 subscales, including total mood disturbance (TMD), *t*(11) = 1.46, *p* = 0.2587 (see Figure 4a for details).

The results suggest that both the Facing and Telenoid conditions produced similar psychological effects, with notable increases in anger–hostility across both conditions. While both conditions showed decreases in other mood-related subscales (FI, F, VA), the magnitude and direction of the changes were relatively consistent, suggesting that both types of interaction (direct vs. Telenoid-mediated) may trigger comparable emotional responses. The lack of significant differences between the two conditions, particularly after FDR correction, suggests that the psychological effects of Telenoid-mediated communication are not markedly distinct from those of face-to-face communication. However, trends toward significance observed in tension–anxiety may warrant further investigation with a larger sample.

#### 3.2.2. HADS (Hospital Anxiety and Depression Scale) Results

Figure 4b presents the results for the Hospital Anxiety and Depression Scale (HADS), comparing both Facing and Telenoid conditions. In the Facing condition, no significant changes were observed in HADS-Anxiety (HADS-A), *t*(11) = 0.98, *p* = 0.4456, HADS-Depression (HADS-D), *t*(11) = 0.00, *p* = 1.000, or HADS-Total, *t*(11) = 1.12, *p* = 0.4020. In contrast, the Telenoid condition showed a trend toward a significant decrease in both HADS-A, *t*(11) = 2.53, *p* = 0.0672, and HADS-D, *t*(11) = 2.71, *p* = 0.0604. Additionally, HADS-Total exhibited a significant reduction, *t*(11) = 3.59, *p* =.0168.

When comparing the two conditions, no significant differences were observed between the two conditions for HADS-A, *t*(11) = 0.91, *p* = 0.4908, HADS-D, *t*(11) = 2.99, *p* = 0.1621, or HADS-Total, *t*(11) = 2.69, *p* = 0.1621.

The results suggest that while no significant changes were found in HADS scores in the Facing condition, the Telenoid condition showed a significant reduction in HADS-Total and trends toward lower HADS-Anxiety and HADS-Depression scores. These findings suggest that operating the Telenoid may help reduce overall anxiety and depression scores, although the lack of significant differences between the two conditions suggests that these effects are subtle and may require larger sample sizes for further validation. The observed trends provide valuable insight into the psychological impact of Telenoid-mediated communication.

#### 3.2.3. SRS-18 (Stress Response Scale-18) Results

Figure 4c presents the results for the Stress Response Scale-18 (SRS-18), comparing the Facing and Telenoid conditions. In the Facing condition, no significant changes were observed in the depression/anxiety (D/A) subscale, *t*(11) = −0.56, *p* = 0.6396, hostility/anger (H/A) subscale, *t*(11) = −1.39, *p* = 0.2866, helplessness, *t*(11) = 0.00, *p* = 1.000, or SRS-18 Total, *t*(11) = −0.68, *p* = 0.5713.

In the Telenoid condition, trends toward a significant reduction were observed in D/A, *t*(11) = 2.45, *p* = 0.0709, and helplessness, *t*(11) = 2.43, *p* = 0.0709. The SRS-18 Total also showed a trend toward a significant decrease, *t*(11) = 2.59, *p* = 0.0667, but no significant change was noted for H/A, *t*(11) = 1.83, *p* = 0.1709.

When comparing the two conditions, no significant differences were found between the two conditions for D/A, *t*(11) = 1.86, *p* = 0.1776, H/A, *t*(11) = 2.17, *p* = 0.1621, helplessness, *t*(11) = 2.09, *p* = 0.1621, or SRS-18 Total, *t*(11) = 2.47, *p* = 0.1621.

Both conditions produced similar stress-related responses, with no significant differences between them. However, the Telenoid condition exhibited trends toward decreased depression/anxiety and helplessness, as well as a reduction in overall stress (SRS-18 Total), suggesting a potential stress-relieving effect when interacting via Telenoid. While these trends did not reach significance, they offer preliminary insights that merit further exploration in studies with larger sample sizes.

### 3.3. The Relationship Between Biological Markers and Psychological Measures

This section explores the correlations between changes in physiological markers (e.g., oxytocin, d-ROMs, cortisol) and psychological measures, including SRS-18, POMS-2 and HADS.

#### 3.3.1. Correlations Between Biological Markers and SRS-18 Changes

Table 1 presents the correlations between changes in hormonal, oxidative, and antioxidant markers and SRS-18 subscales. Significant correlations were observed for oxytocin and d-ROMs:

- Changes in oxytocin levels (pre-post) significantly correlated with both the helplessness subscale, r = 0.43, *p* = 0.035, and the Total SRS-18 score, r = 0.42, *p* = 0.043.
- Changes in d-ROMs (pre-post) significantly correlated with both the helplessness subscale, *r* = 0.61, *p* = 0.0016, and the Total SRS-18 score, *r* = 0.57, *p* = 0.0039.

In contrast, changes in cortisol, GH, and BAP did not show any significant correlations with the SRS-18 subscales or total score, as indicated by non-significant *p*-values (N.S.).

#### 3.3.2. Correlations Between Biological Markers and Other Psychological Measures

Beyond SRS-18 metrics, correlation analyses were also conducted for other psychological measures (POMS-2 and HADS) in relation to changes in biological markers. However, none of these additional correlations reached statistical significance (all *p* > 0.05).

These findings emphasize that oxytocin and d-ROMs are key markers for capturing changes in psychological stress and responses during robot-mediated communication.

## 4. Discussion

### 4.1. Key Findings and Novelty of Telenoid Interaction

This study revealed significant and trending changes in both psychological and biological markers in individuals operating the Telenoid for communication compared to face-to-face interaction. A trending decrease in cortisol levels was observed in the Telenoid condition, a change not seen in the face-to-face session. Furthermore, d-ROMs, a marker of oxidative stress, increased significantly in the face-to-face condition but not in the Telenoid condition. These findings suggest that Telenoid-mediated communication elicits distinct physiological stress responses compared to traditional face-to-face interactions.

The minimalistic design of the Telenoid likely plays a critical role in mitigating stress responses. Unlike face-to-face communication, which involves intricate social cues and emotional dynamics, the Telenoid’s neutral appearance and lack of expressive features may reduce the cognitive and emotional demands on operators. This is consistent with previous findings that simplified humanoid designs reduce social anxiety and the evaluation of apprehension during communication [4]. By eliminating non-verbal cues like facial expressions and gestures, Telenoid minimizes the potential for social judgment and anxiety, particularly in interactions with unfamiliar individuals.

These findings underscore the complexity of stress responses in human–robot interactions and suggest that hormonal markers, such as cortisol, may serve as stable indicators of psychophysiological stress. Blood-based assessments provide a more objective measure of physiological changes compared to subjective questionnaires, capturing nuanced responses that may not be immediately apparent through self-reported data [10,13].

While some trends were observed, such as the trending decrease in cortisol levels in the Telenoid condition, not all results reached statistical significance. This underscores the complexity of stress responses and highlights the importance of larger, more diverse studies in validating these findings.

The results from the SRS-18 questionnaire also showed that participants experienced different dimensions of stress when operating the Telenoid. Trends toward significance were observed in both the depression/anxiety (D/A) and helplessness subscales in the Telenoid condition, suggesting that operating the robot elicited unique psychological responses, likely driven by the novelty and unfamiliarity of the task.

### 4.2. Stress in Different Communication Styles and in Robot Operation

In modern society, communication styles are rapidly shifting from face-to-face interactions to virtual or avatar-mediated interactions, including the use of teleoperated robots like Telenoid. Mediated communication styles, such as video conferencing, have been associated with unique stressors, often termed “Zoom fatigue”, due to cognitive overload and the prolonged virtual engagement they require [5]. Text-based communication has also been found to be more stressful than face-to-face interactions [6]. However, not all mediated communication styles impose similar burdens. Our findings suggest that operating the Telenoid, with its minimalist design and reduced social cues, may alleviate certain stressors commonly found in other mediated settings.

Face-to-face interactions, particularly with unfamiliar partners, are inherently stressful due to the social evaluation and anxiety they can evoke [10,13]. These interactions often require heightened emotional regulation and cognitive effort, reflected in the observed increases in oxidative stress (d-ROMs) after face-to-face sessions. The immediate social presence and the potential for judgment in face-to-face interactions likely account for the elevated psychological and physiological stress markers in this condition.

In contrast, Telenoid-mediated communication likely mitigates these stressors by reducing direct social pressures. This is particularly evident in the trending decrease in cortisol levels and the absence of significant oxidative stress responses in the Telenoid condition. The minimalist design of Telenoid removes the complexities of non-verbal cues, such as facial expressions and gestures, which are often sources of social anxiety [4]. Similar findings have been observed in individuals with autism spectrum disorder (ASD), who are often more comfortable interacting with robotic systems than with humans [23,24,25]. These studies suggest that robotic systems can reduce emotional and social barriers, facilitating communication even in populations prone to heightened stress responses. Our results extend these findings to typical users, highlighting the Telenoid’s potential for creating a less stressful communication environment.

These findings suggest that the physiological and psychological impacts of communication styles are multifaceted. While mediated communication generally involves distinct stress dynamics, the Telenoid’s design offers an opportunity to reduce emotional and cognitive burdens during interactions. Understanding these dynamics is crucial for optimizing robot design and operation protocols to enhance user experiences and well-being.

### 4.3. Interpreting Hormonal and Oxidative Marker Results

The significant correlations between changes in oxytocin and psychological stress scores, particularly in the helplessness subscale and SRS-18 Total, indicate that hormonal changes are closely tied to psychological stress when operating the Telenoid. Additionally, strong correlations between d-ROMs and psychological stress scores suggest that oxidative stress is linked to heightened stress responses during robot operation, particularly in the novel context of Telenoid interactions.

Although cortisol, GH, and BAP did not demonstrate significant changes or correlations in this study, these markers have been widely studied as indicators of stress in various contexts [26,27]. The lack of significant findings may reflect individual variability in stress responses or the specificity of these markers to chronic rather than acute stress conditions [28]. These results highlight the importance of considering a broader range of biological markers in fully understanding the physiological responses to robot-mediated communication.

These findings align with previous studies that associate oxidative stress markers with various stress-related conditions, including aging [12], cardiovascular disease [13], and neurodegenerative diseases like Alzheimer’s disease [29]. The secretion of d-ROMs during stressful conditions may particularly reflect feelings of helplessness, as indicated by their positive correlation with SRS-18 scores.

### 4.4. Pre-Session Values and Novelty Effects

The pre-session values for several measures (e.g., HADS and SRS-18) were higher in the Telenoid condition than in the face-to-face condition. This likely reflects the novelty effect—participants’ initial anxiety and apprehension about operating an unfamiliar teleoperated robot for the first time. Prior research on human–robot interactions and human–computer interfaces suggests that novel systems can trigger heightened anxiety or stress responses, particularly during the first interaction due to factors such as unpredictability or a lack of prior exposure [30,31].

This phenomenon could explain why participants displayed elevated scores in measures like tension–anxiety or mood disturbance before interacting with the Telenoid compared to the more familiar face-to-face interaction. As participants became more accustomed to the robot over the course of the session, their post-session scores reflect a reduction in these psychological responses. Future studies might consider incorporating familiarization sessions with teleoperated robots to mitigate the potential effects of novelty on pre-session measures and allow participants to become accustomed to operating the Telenoid before the experimental session. By mitigating this novelty effect, future research can better isolate the effects of robotic communication from stress induced by unfamiliarity [32].

### 4.5. Limitations

This study has several limitations. First, the small sample size limits the generalizability of its findings. While significant and trending correlations were observed, these results should be interpreted as exploratory and used as a foundation for future research with larger, more diverse samples.

Second, the study focused on young adult participants, who may be more adept at adapting to new technologies, potentially influencing their stress responses. In addition, hormones are secreted differently in younger and older adults [26,27,28,33]. The results, therefore, may not apply to other populations, such as older adults, who may have both different hormonal responses and varying levels of technological proficiency. Future studies should examine a broader demographic range, including older adults and individuals with varying levels of familiarity with new tools, to better understand how these factors influence stress during robot operation.

Third, while some biological markers (e.g., cortisol, GH, BAP) did not show significant changes in response to the Telenoid condition, other markers, such as oxytocin and d-ROMs, demonstrated meaningful correlations with psychological stress. This suggests that certain physiological markers may be more sensitive to the specific type of stress elicited by operating a teleoperated robot. Future research should continue to explore a broader range of biological markers to better understand how different types of stress are reflected in physiological responses during robot-mediated communication.

## 5. Conclusions

This study provides preliminary evidence that operating a teleoperated humanoid robot, such as the Telenoid, can elicit distinct psychological and physiological stress responses. The trending decrease in cortisol levels and the absence of significant increases in oxidative stress markers (d-ROMs) during Telenoid-mediated communication suggest that the robot’s minimalist design may alleviate social pressures and mitigate acute stress compared to face-to-face interactions. Additionally, significant correlations between oxytocin and d-ROMs and psychological stress measures underscore the potential of these markers as objective indicators of psychophysiological stress in human–robot interactions. The findings also highlight the value of reduced social cues in Telenoid-mediated communication, which likely contributed to lower stress levels by minimizing the cognitive and emotional demands on operators. These insights provide practical implications for designing stress-mitigating robotic systems that prioritize user-centered experiences. While cortisol, GH, and BAP did not show significant changes or correlations in this study, their potential utility in chronic stress assessments warrants further investigation. Given the exploratory nature of this research and its small sample size, these findings should be validated in future studies that involve diverse populations and contexts. Larger sample sizes, longitudinal designs, and the inclusion of additional physiological markers will be crucial for understanding the broader applicability of teleoperated robots. This study lays the groundwork for further exploration of the physiological and psychological impacts of robot-mediated communication, offering valuable directions for advancing human–robot interactions in professional, therapeutic, and social domains.

## Figures and Tables

**Table 1:** Correlations Between hormonal/oxidative markers and SRS-18 subscales.

Marker	Helplessness (*r*, *p*)	SRS-18 Total (*r*, *p*)	Other Subscales
Oxytocin	*r* = 0.43, *p* = 0.035	*r* = 0.42, *p* = 0.043	*N.S.*
d-ROMs	*r* = 0.61, *p* = 0.0016	*r* = 0.57, *p* = 0.0039	*N.S.*
BAP	*N.S.*	*N.S.*	*N.S.*
Cortisol	*N.S.*	*N.S.*	*N.S.*
GH	*N.S.*	*N.S.*	*N.S.*

## Data Availability

The research data supporting the reported results and conclusions of this article will be made available by the corresponding author, without undue reservation.
